# Mast Cell and Autoimmune Diseases

**DOI:** 10.1155/2015/246126

**Published:** 2015-04-05

**Authors:** Yunzhi Xu, Guangjie Chen

**Affiliations:** Department of Immunology and Microbiology, Shanghai Jiao Tong University School of Medicine, Shanghai Institute of Immunology, Shanghai 200025, China

## Abstract

Mast cells are important in innate immune system. They have been appreciated as potent contributors to allergic reaction. However, increasing evidence implicates the important role of mast cells in autoimmune disease like rheumatoid arthritis and multiple sclerosis. Here we review the current stage of knowledge about mast cells in autoimmune diseases.

## 1. Introduction

If the immune system fails to recognize self- from non-self-molecules, self-reactive lymphocytes can be activated by innate immune cells and lead to an autoimmune response [1]. Genetics, hormonal influences, and environment play important roles in autoimmune diseases. Some of the factors have been identified [2–4]. However, the specific determinants that initiate an autoimmune response and allow it to be sustained and cause pathology are still unknown. Autoimmune diseases and allergic diseases share important features. Both of them are the result of “hypersensitive” immune responses directed toward inherently harmless antigens [5]. Besides, many diseases models that we now know are regarded as autoimmune diseases, such as “experimental allergic” neuritis, encephalomyelitis, orchitis, uveitis, and glomerulonephritis [6]. It is accepted that the cells of the adaptive immune system are the directors of autoimmune responses [7]. In addition, innate immune cells are critical for sustaining the response that leads to pathology [8–13].

Mast cells (MCs) are first described by Paul Ehrlich in 1878 [1]. They have been viewed as effectors in IgE-mediated allergic or antiparasitic responses; however, researches in the last two decades have found that MCs are also involved in innate immunity and inflammation by releasing a large array of inflammatory mediators [14, 15]. These mediators include compounds such as histamine and MC specific proteases prestored in cytoplasmic secretory granules (SGs) and newly synthesized lipid mediators such as leukotrienes or prostaglandins or a variety of cytokines, chemokines, and growth factors [16].

The idea that MCs are involved in the initiation and sustaining events of autoimmunity is based on abundant data from studies of both human disease and animal models [17–19].

## 2. Mast Cells

MCs were discovered by Friedrich von Recklinghausen in 1863 and named by Paul Ehrlich in 1878 [20]. Connective tissue is derived from undifferentiated mesenchymal cells. During the first 100 years after the discovery, it was believed that MCs were a component of connective tissue, functioned, and died within connective tissue [21]. Furthermore, MCs complete differentiation in connective tissue [21]. Until the 1980s,* in vivo* and* in vitro* evidence showed that MCs originate from hematopoietic stem cells, but the mast cell-committed precursors (MCPs) have not been identified [21, 22]. In the work of Chen et al., MCPs in the bone marrow of adult mice were identified. They are identified by the phenotype Lin^−^ c-Kit^+^ Sca-1^−^ Ly6c^−^Fc*ε*RI*α* ^−^ CD27^−^ *β*7^+^ T1/ST2^+^ [23]. In addition, the experiment strongly suggests that MCPs are the progeny of multipotential progenitors (MPPs) other than common myeloid progenitors or granulocyte/macrophage progenitors [23].

Development of MCs from MPPs does not need cell division [21]. It is known that MCs leave the bone marrow as immature cells and they mature via abundant cytokines in the local tissue microenvironment [20, 24]. For example, nerve growth factor (NGF) is well known as an important MCs growth factor [25]. However, MCs show plasticity [20]. Moreover, mature MCs show extensive proliferation potential [21].

The granules of MCs can be stained metachromatically purple with Toluidine Blue and it is routine staining for the demonstration of MCs [20, 26]. MCs are defined as connective tissue mast cells (CTMCs) and mucosal mast cells (MMCs) by the histamine, cytokines, and proteolytic enzyme which MCs store [20]. In addition to innate and acquired immunity, MCs play important role in bacterial infection and autoimmunity [24, 27, 28]. MCs can secrete the contents of preformed cytoplasmic secretory granules (SGs) while encountering certain stimulants. For MCs, this process is fundamental to their role in innate and acquired immunity [29]. Various molecules are able to activate MCs.

## 3. Interactions between Mast Cells and Other Cells

MCs can work with other cells like T and B lymphocytes to enhance activation and migration by cell-cell interactions or secreted products [18, 19]. Recently, the role of the interactions between mast cells and other cells in autoimmune diseases is becoming apparent [30].

### 3.1. Interaction among Mast Cells, T Regulatory Cell (Treg), and Th17 Cells

**Figure 1:**
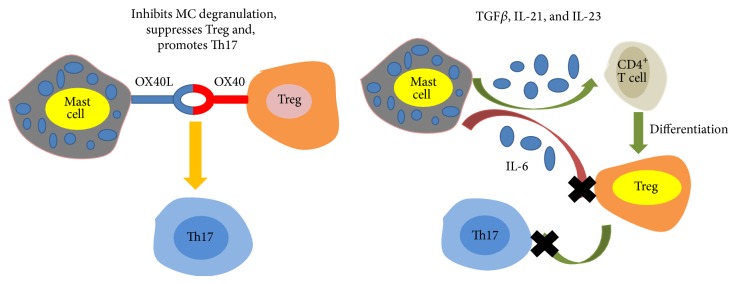
Direct cell interaction between mast cells and T cells.

Treg cells are defined as CD4^+^CD25^+^FoxP3^+^ and are known to suppress T effector cell response. Thus Treg cells can induce tolerance and control autoimmunity. MCs and Treg cells constitutively express OX40L and OX40, respectively. Therefore, mast cell-Treg cell interactions are in an OX40-dependent way. Gri et al. found that Treg cells directly inhibited Fc*ε*RI-dependent MC degranulation through cell-cell contact requiring OX40-OX40L interaction [31] (Figure 1). Kashyap's group showed that coculture with Treg enhanced cytokines production by MCs [32]. In addition, MCs can also suppress Treg activity in an OX40L-independent way [30]. However, the relationship between MCs and Treg cells needs to be further explored in autoimmunity.

Th17 cells are CD4^+^ T cells. At the meantime, they are defined by the expression of the transcription factor ROR*γ*t and cytokines IL-17. As Th1 cells, Th17 cells are involved in the mouse models of MS and RA. The combination of TGF*β*, IL-6, IL-21, IL-23, and IL-1*β* contributes to the differentiation of Th17 from a naïve CD4^+^ T cell. TGF*β* is essential for the development of Treg cells, but it is inhibited by IL-6. MCs can express TGF*β*, IL-6, IL-21, and IL-23 under some condition and promote Treg and Th17 cell differentiation and plasticity [30]. It is interesting that MCs counteract Treg cells suppression through IL-6 and OX40-OX40L axis towards Th17 cell differentiation [33] (Figure 1).

### 3.2. Interaction between Mast Cells and B Cell

**Figure 2:**
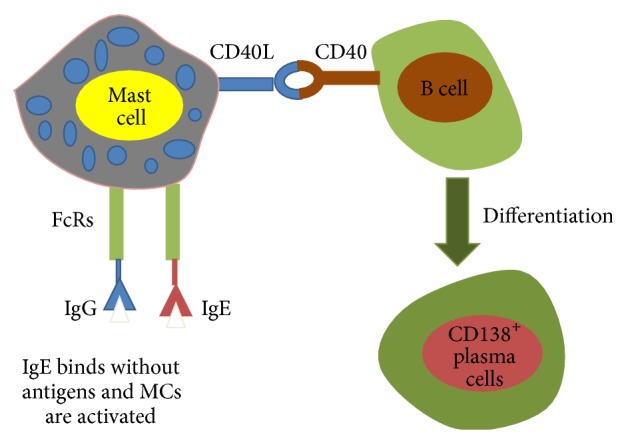
Direct cell interaction between mast cells and B cells.

MCs express a variety of B cell-modulating molecules and immunoglobulin (Ig) receptors [30]. MC FcRs include IgE and IgG receptors [34]. Depending upon the type of MCs, IgG-antigen complexes may activate MCs [34]. Conversely, the coengagement of IgG and IgE receptors inhibits cells activation [34]. Increasing data has been established indicating that MCs play critical roles in IgG-dependent tissue-specific autoimmune diseases [34]. Low amounts of MCs are effective in influencing B cell survival and proliferation* in vitro* through cell-cell contact and MC-derived IL-6 expression whatever state the MC activation is in [35]. Furthermore, MCs can promote B cells to differentiate into CD138^+^ plasma cells secreting IgA and it is dependent on CD40-CD40L expressed on B cells and MCs, respectively [35] (Figure 2).

## 4. MCs and Autoimmune Diseases

It is well known that T cells are important in directing and initiating the immune response in the target tissues [30]. In addition, other cells also play an important role in aggravating the inflammatory damage [30]. Furthermore, there are several examples of MCs association with autoimmune diseases including multiple sclerosis (MS), rheumatoid arthritis (RA), insulin-dependent diabetes mellitus (IDDM), bullous pemphigoid, chronic idiopathic urticaria, and experimental vasculitis [36–39]. Here we take MS, RA, IDDM, and chronic urticaria (CU) for example and summarize the role of MCs in the autoimmune diseases.

### 4.1. MCs and MS

Mostly, the interest in the role of MCs in the initiation and propagation of autoimmune disease comes from studies on MS [40].

MS is a progressive demyelinating disease. Widespread inflammatory lesions present in the brain and spinal cord of patients with MS [30]. The symptoms of MS contain visual disturbances, bowel and bladder incontinence, and sensory and motor dysfunction [30]. Furthermore, patients with MS are found to lose memory, impair attention, and slow information processing [41, 42]. Experimental autoimmune encephalomyelitis (EAE) is a murine model of MS. Similar to MS, the symptoms of EAE resulted from breach of the blood-brain barrier (BBB) which allows inflammatory cells to infiltrate into the central nervous system (CNS) and destruct myelin and oligodendrocytes [30]. CD4^+^ T cells, including IFN-*γ*-secreting T helper 1 cells (Th1), IL-17-producing T helper 17 cells (Th17), and IL-9-producing T helper 9 cells (Th9), contribute to the pathogenic autoimmune response in EAE [43]. However, the roles of these cells in MS are still unclear [44].

There are MCs in the leptomeninges, the choroid plexus, thalamus, hypothalamus, and median eminence [24]. Similar to CTMCs and MMCs, brain mast cells (BMCs) can be identified morphologically by Toluidine Blue staining mostly. Moreover, histamine fluorescence with *ο*-phthaldialdehyde is able to show BMCs in the leptomeninges, thalamus, and hypothalamus. And histamine immunohistochemistry can show BMCs in the median eminence [45–48]. However, many BMCs are stained with Sudan Black which is distinct from CTMCs or MMCs [20]. Additionally, the ultrastructural appearance of activated BMCs is different from that of CTMCs because it is primarily characterized by intragranular changes without typical compound exocytosis [49, 50]. They may regulate vascular permeability and inflammatory cell entry in the brain parenchyma [51]. Moreover, there is interaction between functional MCs and neuron in the brain and it can mediate neuroinflammation.

Kruger et al. have observed MCs within the demyelinated plaques in the brains of 7 patients with MS [26]. Moreover, MCs were found mostly located in close connection with small vessels [26]. The data suggest that MCs playing a role in MS have continued to accumulate [30]. It is reported that mast cell deficient mice fail to develop EAE [52]. As in MS, an increase of MCs is also found at sites of inflammatory demyelination in the brain and spinal fluid of EAE [53]. MCs are associated with Fc*ε*R, the histamine-1 (H1) receptor, and tryptase [24]. Elevated levels of tryptase are present in the cerebrospinal fluid of MS patients and gene array analyses of MS reveal overexpression of genes encoding Fc*ε*R, H1 receptor, and tryptase [24, 54]. BMCs do not express their surface growth factor (c-kit) receptor normally but do so during EAE [55]. Several studies reveal that mast cell-derived mediators can increase BBB permeability [56, 57]. Products produced by MCs can enter neurons and this indicates a new brain-immune system [58]. Rat BMCs can produce tumor necrosis factor (TNF) and TNF take part in both brain inflammation and increased vascular permeability [59, 60]. An increased mast cell tryptase in the cerebrospinal fluid (CSF) of MS patients can activate peripheral mononuclear cells to secrete TNF, IL-6, and IL-1 and stimulate protease-activated receptor (PAR) which leads to microvascular leakage and widespread inflammation [54, 61, 62]. Besides, human MCs will secrete matrix metalloproteinase- (MMP-) 9 and IL-6 while contacting activated T cells [63]. So we proposed that MCs may be an underestimated contributor to the demyelinating process of MS.

All in all, MCs participate in the pathogenesis of MS in many different ways [24]. Firstly, they release cytokines/chemokines to recruit and activate T cell/macrophage after stimulation. Secondly, MCs present myelin antigen to T cell. Furthermore, MCs disrupt the BBB to allow activated T cells to infiltrate to brain and target in myelin basic protein (MBP). What is more, MCs damage myelin and then release fragments resulting in stimulating secretion of tryptase. In turn, it enhances demyelination and induces further inflammation through stimulation of PAR possibly. As a result, MCs can be a possible therapeutic target for MS.* In vitro*, on one hand, mast cell proteases degrade myelin protein, while on the other hand, myelin stimulates mast cell degranulation directly [64, 65]. Therefore, treatment with inhibitors of mast cell degranulation may be a good way to inhibit MS. Dimitriadou et al. found that hydroxyzine was able to inhibit EAE [66].

### 4.2. MCs and RA

RA is a systemic and chronic inflammatory disease that affects about 1% of the population worldwide [30, 67]. After decades of research, we have found that T and B lymphocytes, neutrophils, monocytes, and vascular endothelium play the roles in RA [67]. However, the pathogenesis and mechanism of RA are still unclear [67]. Rodent models of autoimmune diseases are of great use to study the pathogenic process of diseases. There are a number of models of RA including K/BxN, adjuvant-induced and pristane models, but the streptococcal cell wall (SCW) arthritis in rat and the collagen-induced arthritis (CIA) in mice are the most widely used [67].

Lee et al. found that W/Wv and Sl/Sld, which are deficient in MCs, were resistant to development of joint inflammation. They proposed that MCs may serve as a cellular link among numerous components in inflammatory arthritis [68]. What is interesting is that MCs are normally expressed in the synovial compartments of healthy people but increased in RA patients [69]. The number of MCs increases 5- to 24-fold in affected joints in human RA when compared to the number of those in normal joints [69]. It is also found that MCs number expand more than 3-fold in multiple animal models of RA [70–72]. Besides, the cytokines and proteases which are produced by MCs are involved in the pathogenic process of RA, particularly TNF, IL-1*β*, IL-17, and tryptase [30, 73]. Tryptase is a preformed mast cell-specific protease and is thought to lead to the inflammatory response by working with heparin to induce the neutrophils and synovial fibroblasts to release cytokines [74]. Tryptase can also directly activate synovial fibroblasts by interacting with the protease-activated receptor 2 (PAR2) to express more proteases that degrade cartilage and bone [75, 76].

Matsumoto and Staub's group found that RA may be associated with the enzyme glucose-6-phosphate isomerase (GPI) [77]. K/BxN mice produce autoantibodies that can recognize GPI. The antibodies aggregate with GPI, and then immune complex is deposed on the surface of the articular cavity to initiate a signaling cascade including MCs. Cytokines such as IL-1 and IL-17A are also involved [73, 78]. The serum from K/BxN mouse causes similar inflammatory arthritis in a wide range of mouse strains, but Kit^W/W-v^ mouse deficient in MCs resistant to autoimmune inflammatory arthritis was induced by injection of sera from K/BxN mouse. If the MCs are reconstituted, the sensitivity would be restored [68]. Kit^W-sh^ mice deficient in MCs are sensitive to autoimmune inflammatory arthritis induced by injection of sera from K/BxN mouse and mast cell-reconstituted Kit^W-sh^ mice are still susceptible to arthritis induced by sera from K/BxN mouse [79].

MCs accumulate in the synovial tissues and fluids of patients with rheumatoid arthritis and produce inflammatory mediators [1]. In addition to the degranulation in the articulate cavity after antibody administration, the activation of MCs through the IgG immune complex receptor Fc*γ*RIII can precipitate the initiation of inflammation within the joint through the production and release of IL-1 [68, 80]. Stem cell factor (SCF) is essential for mast cell survival and development* in vitro* [1]. Furthermore, TNF-*α* derived from MCs can induce fibroblasts to produce SCF, the ligand for the CD117/c-Kit receptor [81, 82]. SCF increases the recruitment of MCs and creates an amplification loop [81, 82].

### 4.3. MCs and IDDM

Insulin-dependent diabetes mellitus (IDDM) is also called type I diabetes. IDDM is a chronic metabolic disorder that develops in two discrete phases and is mediated in part by CD8^+^ T cells [19, 83]. In the process of IDDM, various leukocytes invade the pancreatic islets and lead to insulitis. Then the insulin-producing *β* cells of the pancreas are destructed and lead to hyperglycemia [19]. Furthermore, IDDM is commonly associated with immune-mediated damage [84]. There are several rodent models of IDDM. In susceptible rodents, small dose of streptozotocin induces insulinopenic diabetes in which immune destruction plays the role, as in human type I diabetes [85]. In addition, the nonobese diabetic (NOD) mouse and biobreeding (BB) rat are the two most commonly used animals that spontaneously develop diseases with similarities to human type I diabetes [85].

Normally, MCs locate within the pancreatic ducts and are close to the pancreatic islets [86]. A lot of studies have found a striking increase in the frequency of MCs in the acinar parenchyma in inflammatory disease of pancreas [86–88]. Besides, MCs produce various mediators which are able to affect the development of IDDM. For example, leukotriene B4 (LTB4), which is released by MCs and may be important for recruitment or retention of autoreactive T cells in the target organ, is found increased in type I diabetes [89]. What is the most important is that Geoffrey et al. discovered more MCs in the pancreatic lymph nodes of lymphopenic diabetic BB rats before disease onset [36]. As a result, there is suspicion that MCs are involved in IDDM.

### 4.4. MCs and CU

Chronic urticaria (CU) is a distressing disorder that adversely impacts the quality of life, but its pathogenesis is not delineated well [90]. An autoimmune subset of chronic spontaneous urticaria is increasingly being recognized internationally based on laboratory and clinical evidence that has accrued over the last 20 years [91]. In 1983, Leznoff et al. suggested that urticaria should be considered autoimmune [92]. Gruber et al. detected functional anti-IgE antibodies and proposed that these could be the cause of urticarial wheals [93]. And now it is well recognized that about 30–50% CU patients have circulating functional autoantibodies against the high-affinity IgE receptor or against IgE [94]. Besides, CU is associated with various autoimmune diseases [95].

Urticaria is triggered by inappropriate activation and degranulation of dermal mast cells. And the cellular contents released by MCs prime the immediate phase of inflammation, resulting in a lymphocyte and granulocyte mediated hypersensitivity reaction [96]. In turn, the infiltrating inflammatory cells produce more proinflammatory mediators to recruit and activate other cells and extend the host response [96]. It lowers the reactive threshold of MCs to induce stimuli and promotes the maintenance of susceptibility to urticaria [90]. It provides an explanation for Smith's discovery that MCs numbers remain unaltered [97]. Bossi et al. evaluated permeabilizing activity of sera from CU patients and healthy people by measuring serum-induced degranulation of two MC lines (LDA2 and HMC-1) [98]. They discovered that almost all the CU patients sera promoted degranulation of MCs and 17/19 mast cell supernatant from HMC-1 and SNs from LAD2 incubated with CU sera increased endothelia permeability [98]. It is said that histamine released from MCs is the major effector on pathogenesis [94]. Bossi et al. also found that endothelial cell leakage was prevented by antihistamine [98].

## 5. Conclusion

It is clear that MCs play an important role in autoimmune diseases. In conclusion, MCs can worsen disease by a number of mediators and counteracting Treg cells function. In the mouse models of RA and MS, MCs promote inflammation in the same way like TNF.

MCs can be a new treatment target in the autoimmune diseases because of their pivotal position in the inflammation process. The therapeutic strategies focus on three aspects as follows: (1) at the level of the molecules produced by MCs, (2) at the level of MCs activation, and (3) at the level of MC proliferation [99]. The study of Saso demonstrated that MCs can be inhibited through the action of an Fc*ε*–Fc*γ* fusion protein engineered to engage human Fc*γ*RIIb with high affinity. This study suggests that analogous fully human Fc*ε*–Fc*γ* tandem Fc biologic has potential as a potent and selective inhibitor of cellular activation and degranulation and thus represents a promising approach in treating mast cell and basophil-mediated pathogenesis [100]. Masitinib, a selective oral tyrosine kinase inhibitor, effectively inhibits the survival, migration, and activity of MCs. Vermersch's group assessed the masitinib treatment in patient with progressive MS and the data suggested that masitinib is of therapeutic benefit to MS patients [101].

Cpa3^Cre/+^ mice are a strain deficient in MCs. In spite of a great deal of evidence of the involvement of MCs in the autoimmune disease models, using Cpa3^Cre/+^ mice in study did not find an active role of MCs in both the K/BxN serum transfer model of RA and the EAE model of MS [102]. Besides, Gutierrez et al. found that IDDM in NOD mice was unaffected by mast cell deficiency [103]. Therefore, the research about the roles of MCs in autoimmune diseases remains a matter of great debate and ought to be further studied, which is important for creating new MC targeted therapies [5].

## Abbreviations

BB: Biobreeding rat
BBB: Blood-brain barrier
BMCs: Brain mast cells
CIA: Collagen-induced arthritis
CNS: Central nervous system
CSF: Cerebrospinal fluid
CTMCs: Connective tissue mast cells
CU: Chronic urticaria
EAE: Experimental autoimmune encephalomyelitis
GPI: Glucose-6-phosphate isomerase
H1: Histamine-1
IDDM: Insulin-dependent diabetes mellitus
Ig: Immunoglobulin
LTB4: Leukotriene B4
MBP: Myelin basic protein
MCPs: Mast cell-committed precursors
MCs: Mast cells
MMCs: Mucosal mast cells
MMP: Matrix metalloproteinase
MPPs: Multipotential progenitors
MS: Multiple sclerosis
NGF: Nerve growth factor
NOD: Nonobese diabetic mouse
PAR: Protease-activated receptor
PAR2: Protease-activated receptor 2
RA: Rheumatoid arthritis
SGs: Secretory granules
SCW: Streptococcal cell wall
Th1: T helper 1 cells
Th17: T helper 17 cells
Th9: T helper 9 cells
TNF: Tumor necrosis factor
Treg: T regulatory cell.
